# LncRNA TDRKH-AS1 promotes breast cancer progression via the miR-134-5p/CREB1 axis

**DOI:** 10.1186/s12967-023-04640-3

**Published:** 2023-11-26

**Authors:** Yuqin Ding, Yuting Huang, Fanrong Zhang, Lijie Gong, Chenlu Liang, Kaijing Ding, Xiangming He, Xiaowen Ding, Yiding Chen

**Affiliations:** 1 Department of Breast Surgery, The Second Affiliated Hospital, Zhejiang University School of Medicine, Hangzhou, 310009 Zhejiang China; 2 Department of Breast Surgery, Zhejiang Cancer Hospital, Institute of Cancer and Basic Medicine (IBMC), Chinese Academy of Sciences, Hangzhou, 310022 Zhejiang China; 3 Wenzhou Medical University, Wenzhou, 325000 Zhejiang China; 4 Department of Child Psychology, Affiliated Mental Health Center & Hangzhou Seventh People’s Hospital, Zhejiang University School of Medicine, Hangzhou, 310013 Zhejiang China

**Keywords:** Breast cancer, lncRNA, TDRKH-AS1, Competing endogenous RNA, Progression

## Abstract

**Background:**

Breast cancer (BC) is a prevalent malignancy with complex etiology and varied clinical behavior. Long non-coding RNAs (lncRNAs) have emerged as key regulators in cancer progression, including BC. Among these, lncRNA TDRKH-AS1 has been implicated in several cancers, but its role in BC remains unclear.

**Methods:**

We conducted a comprehensive investigation to elucidate the role of TDRKH-AS1 in BC. Clinical samples were collected from BC patients, and BC cell lines were cultured. Bioinformatics analysis using the starBase database was carried out to assess TDRKH-AS1 expression levels in BC tissue samples. Functional experiments, including knockdown, colony formation, CCK-8, Transwell, and wound-healing assays, were conducted to determine the role of TDRKH-AS1 in BC cell proliferation and invasion. Luciferase reporter and RIP assays were used to examine the interactions between TDRKH-AS1 and miR-134-5p. In addition, the downstream target gene of miR-134-5p, cAMP response element-binding protein 1 (CREB1), was identified and studied using various methods, including RT-qPCR, immunoprecipitation, and rescue experiments. In vivo experiments using mouse tumor xenograft models were conducted to examine the role of TDRKH-AS1 in BC tumorigenesis.

**Results:**

TDRKH-AS1 was found to be significantly upregulated in BC tissues and cell lines. High TDRKH-AS1 expression correlated with advanced BC stages and worse patient outcomes. Knockdown of TDRKH-AS1 led to decreased BC cell proliferation and invasion. Mechanistically, TDRKH-AS1 acted as a sponge for miR-134-5p, thereby reducing the inhibitory effects of miR-134-5p on CREB1 expression. Overexpression of CREB1 partially rescued the effects of TDRKH-AS1 knockdown in BC cells. In vivo studies further confirmed the tumor-promoting role of TDRKH-AS1 in BC.

**Conclusions:**

Our study unveiled a novel regulatory axis involving TDRKH-AS1, miR-134-5p, and CREB1 in BC progression. TDRKH-AS1 functioned as an oncogenic lncRNA by promoting BC cell proliferation and invasion through modulation of the miR-134-5p/CREB1 axis. These findings highlighted TDRKH-AS1 as a potential diagnostic biomarker and therapeutic target for BC treatment.

**Supplementary Information:**

The online version contains supplementary material available at 10.1186/s12967-023-04640-3.

## Introduction

Breast cancer (BC), a prevalent malignancy that affects the breast tissue, is caused by multiple factors including genetic alterations, environmental influences, and personal lifestyle decisions [1]. BC commonly presents as a lump in the breast or an abnormal mammogram result, while other symptoms may include breast pain, nipple discharge, or skin changes [2, 3]. Early detection through regular screening and self-examination can significantly increase the chances of successful treatment and long-term survival of BC patients [4]. However, in some cases, particularly aggressive forms of BC may lead to the development of resistance to traditional treatments such as surgery, radiotherapy, chemotherapy, and hormone therapy. Thus, researchers are actively developing novel therapeutic approaches, such as targeted therapies and immunotherapies, to help improve patient outcomes and prevent recurrence [5–7]. Overall, due to its prevalence and potentially lethal consequences, BC remains a significant public health concern and a major research focus.

Long non-coding RNAs (lncRNAs) play critical roles in regulating gene expression, chromatin remodeling, and modulating protein activity [8]. Initially considered to be “transcriptional noise”, lncRNAs have increasingly been implicated in many diseases, such as cancer, via multiple mechanisms [9]. Many lncRNAs have been shown to be dysregulated in various cancers [10]. TDRKH-AS1 is a lncRNA that is transcribed from the antisense strand of the TDRKH gene locus and plays a crucial role in the regulation of cancer cell proliferation, apoptosis, and migration. TDRKH-AS1 is located at chromosomal position 1q21.3 and contains three spliceosomes [11]. Previous studies have indicated that TDRKH-AS1 from trophoblast-secreted vesicles may contribute to preeclampsia by inducing endothelial cell injury and inflammation through oxidative stress [12]. Importantly, a high-throughput computational study recently reported that the TDRKH-AS1 copy number was associated with the survival rate of lung adenocarcinoma (LUAD) patients [13]. TDRKH-AS1 has also been shown to be overexpressed in colorectal cancer (CRC) tissues and significantly associated with the malignancy features of CRC, as well as poor prognoses [11]. In addition, upregulation of TDRKH-AS1 has been reported in hepatocellular carcinoma (HCC) tissues, while its knockdown has been shown to suppress HCC progression [14]. Understanding the molecular mechanisms of oncogenic lncRNAs such as TDRKH-AS1 is essential for the development of novel clinical strategies for cancer diagnosis, prognosis and treatment. Based on these previous studies, TDRKH-AS1 may be a potential therapeutic target for future cancer treatments.

Here, we found that TDRKH-AS1 was significantly upregulated in BC tissue samples. In addition, we demonstrated that overexpression of TDRKH-AS1 promoted cell proliferation and induced the epithelial-mesenchymal transition (EMT) in BC cell lines. Further analysis revealed that TDRKH-AS1 was predominantly expressed in the cytoplasm of BC cells, and functioned as a competitive endogenous RNA (ceRNA) for miR-134-5p, thereby reducing the inhibitory effects of miR-134-5p on its target gene cAMP response element-binding protein 1 (CREB1). Together, our findings indicate that TDRKH-AS1 could potentially serve as a valuable biomarker for predicting prognosis and guiding treatment decisions in BC patients.

## Results

### The lncRNA TDRKH-AS1 is overexpressed in BC tissues

**Fig. 1:**
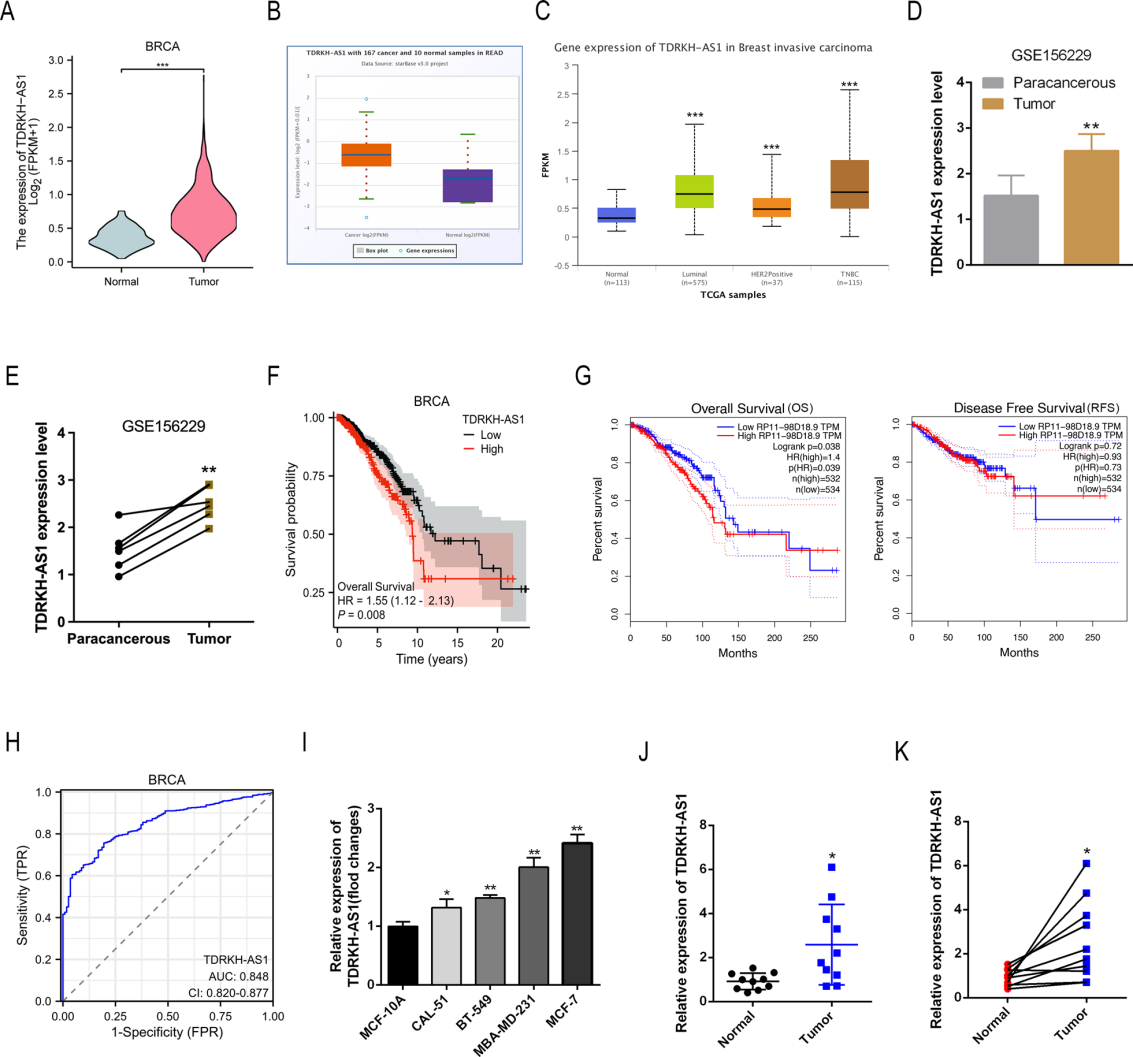
TDRKH-AS1 is overexpressed in BC tissues and cell lines. **A**, **B** The TCGA and starBase database results showed that TDRKH-AS1 expression was obviously upregulated in BC tissues compared with normal tissues. ^∗∗∗^*P* < 0.001 vs. Normal group. **C** The UALCAN database results showed that TDRKH-AS1 expression was obviously upregulated in in different BC molecular subtypes including luminal, HER2-positive and TNBC compared with normal tissues. ^∗∗∗^*P* < 0.001 vs. Normal group. **D**, **E** The GEO database displayed that TDRKH-AS1 expression was upregulated in BC tissues. ^∗∗^*P* < 0.01 vs. Paracancerous group. **F** The OS curves of BC patients were generated by Kaplan–Meier analysis from TCGA database. Elevated TDRKH-AS1 expression was associated with a poor prognosis in patients. **G** GEPIA database was used to analyze the impact of TDRKH-AS1 expression levels on clinical prognosis, including OS and RFS. **H** The ROC curves analysis showed that TDRKH-AS1 could be a latent discriminator between breast cancer and normal tissues. **I** Expression levels of TDRKH-AS1 in BC cell lines were detected by RT-qPCR. ^∗^*P* < 0.05 ^∗∗^*P* < 0.01 vs. control group. **J**, **K** RT-qPCR detected expression levels of TDRKH-AS1 in clinical samples. ^∗^*P* < 0.05 vs. Normal group

First, we analyzed the lncRNA expression profiles of BC samples from the TCGA database using the starBase database, and found that TDRKH-AS1 expression levels were markedly elevated in BC tissues (Fig. 1A, B). In addition, using the UALCAN database, we found that TDRKH-AS1 expression was significantly higher in luminal BC, HER2-positive BC, and triple-negative BC (TNBC) samples than normal samples. However, no significant difference in TDRKH-AS1 expression levels was observed among these three BC groups (Fig. 1C). Analysis of six paired tissues from the GEO database (dataset: GSE156229) confirmed that TDRKH-AS1 was upregulated in BC tumor tissue (Fig. 1D, E). Kaplan–Meier analysis revealed that high TDRKH-AS1 expression levels were associated with low survival rates in BC patients (Fig. 1F). Furthermore, using the GEPIA database to analyze the relationship between TDRKH-AS1 expression levels and clinical prognosis, including OS and RFS, we found that TDRKH-AS1 expression levels were significantly correlated with OS, while no statistically significant differences in RFS were observed (Fig. 1G). ROC curve analysis confirmed that TDRKH-AS1 expression levels could serve as a potential diagnostic biomarker for BC, with an area under the curve (AUC) value of 0.848 (Fig. 1H). In addition, we found that TDRKH-AS1 expression levels were higher in four BC cell lines (CAL-51, BT-549, MBA-MD-231 and MCF-7) than one normal epithelial cell line (MCF-10A) (Fig. 1I). Finally, RT-qPCR analysis of ten BC tissue and matched non-cancerous tissue samples revealed that TDRKH-AS1 was significantly upregulated in BC tissue compared to non-cancerous tissue (Fig. 1J, K).

To examine the potential clinical significance of TDRKH-AS1 in BC, we conducted a correlation analysis using data from the TCGA database to investigate the correlation between clinical characteristics of BC and TDRKH-AS1 expression levels. Our findings revealed a significant correlation between TDRKH-AS1 expression and the M stage of BC patients, suggesting that TDRKH-AS1 may have prognostic value as a biomarker for BC (Table 1). Together, our findings suggested that TDRKH-AS1 was upregulated in BC and that its overexpression could potentially play a key role in progression of the disease.

**Table 1:** Correlation of TDRKH-AS1 with clinicopathological characteristics in The Cancer Genome Atlas (TCGA) cohort

Characteristic	Low expression of TDRKH-AS1	High expression of TDRKH-AS1	P
n	541	542	
T stage, n (%)			0.087
T1	155 (14.4%)	122 (11.3%)	
T2	298 (27.6%)	331 (30.6%)	
T3	67 (6.2%)	72 (6.7%)	
T4	20 (1.9%)	15 (1.4%)	
N stage, n (%)			0.892
N0	254 (23.9%)	260 (24.4%)	
N1	179 (16.8%)	179 (16.8%)	
N2	62 (5.8%)	54 (5.1%)	
N3	38 (3.6%)	38 (3.6%)	
M stage, n (%)			0.031^*^
M0	468 (50.8%)	434 (47.1%)	
M1	5 (0.5%)	15 (1.6%)	
Age, meidan (IQR)	58 (49, 67)	58 (48.25, 67)	0.580

^∗^P < 0.05

### Knockdown of TDRKH-AS1 impedes BC cell proliferation and invasion

**Fig. 2:**
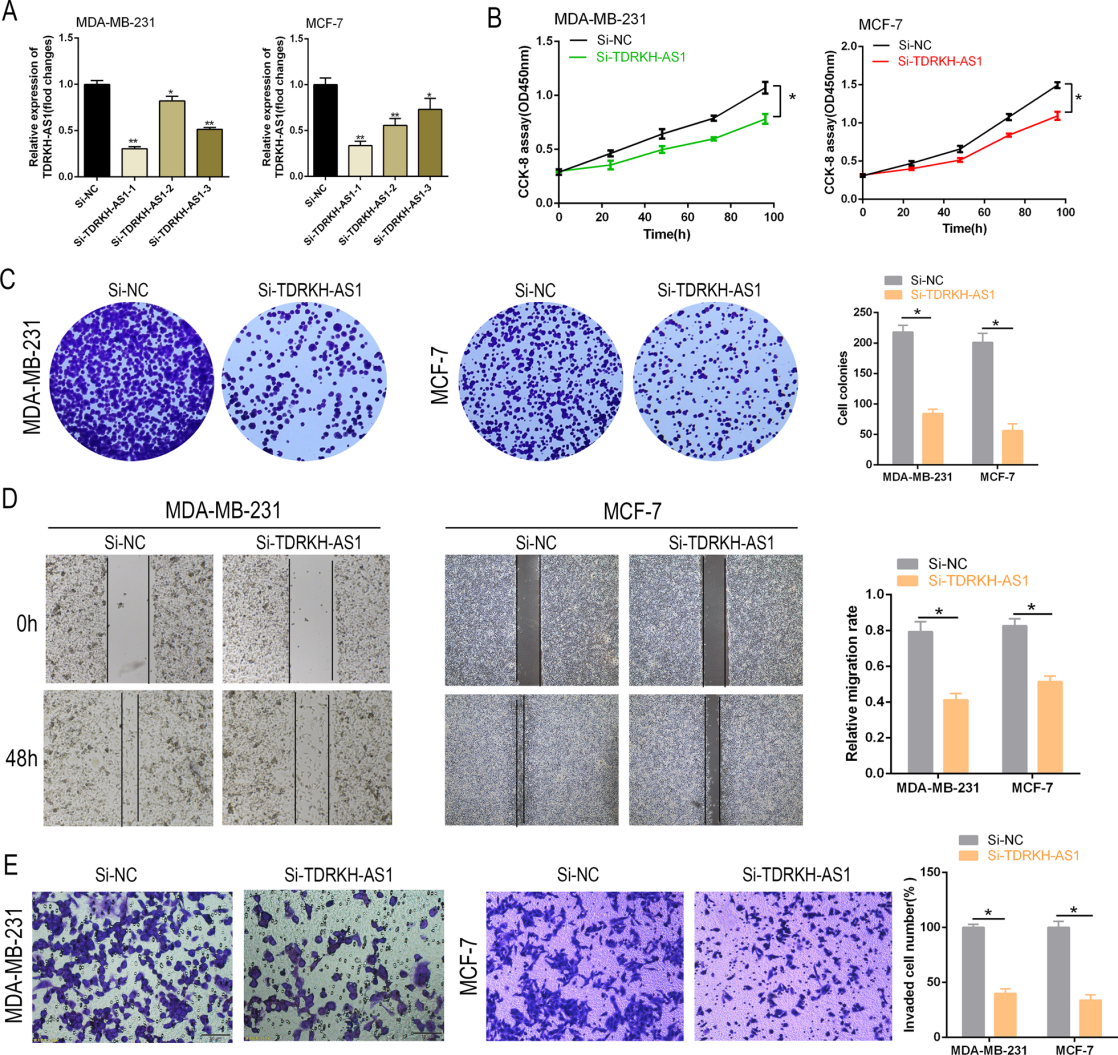
TDRKH-AS1 knockdown suppresses BC cell proliferation and invasion in vitro. **A** TDRKH-AS1 expression was reduced in MDA-MB-231 and MCF-1 cells using siRNAs. **B**, **C** The results of CCK-8 and colony formation assays demonstrated that the knockdown of TDRKH-AS1 inhibited the proliferation of breast cancer cells with a *P* value < 0.05. **D**, **E** Wound healing and transwell assays were conducted to assess the effect of si-TDRKH-AS1 on the invasion ability of breast cancer cells. The results showed a significant decrease in cell invasion ability in the knockdown group compared to the NC group. ^∗^*P* < 0.05 vs. NC group

Next, we silenced TDRKH-AS1 expression in MCF-7 and MDA-MB-231 cells using three different siRNAs specifically targeting TDRKH-AS1 (si-TDRKH-AS1#1, #2, #3) to determine the role of TDRKH-AS1 in BC progression. RT-qPCR analysis indicated satisfactory knockdown of TDRKH-AS1, and si-TDRKH-AS1-1 was selected for subsequent experiments (Fig. 2A, B). Colony formation and CCK-8 assays revealed that knockdown of TDRKH-AS1 inhibited cell growth (Fig. 2C). Similarly, the Transwell and wound-healing assays showed that knockdown of TDRKH-AS1 reduced cell invasion in BC cells (Fig. 2D, E). Our findings indicated that TDRKH-AS1 was involved in the regulation of cell proliferation and invasion in BC cells.

### TDRKH-AS1 acts as a sponge for miR-134-5p in BC

**Fig. 3:**
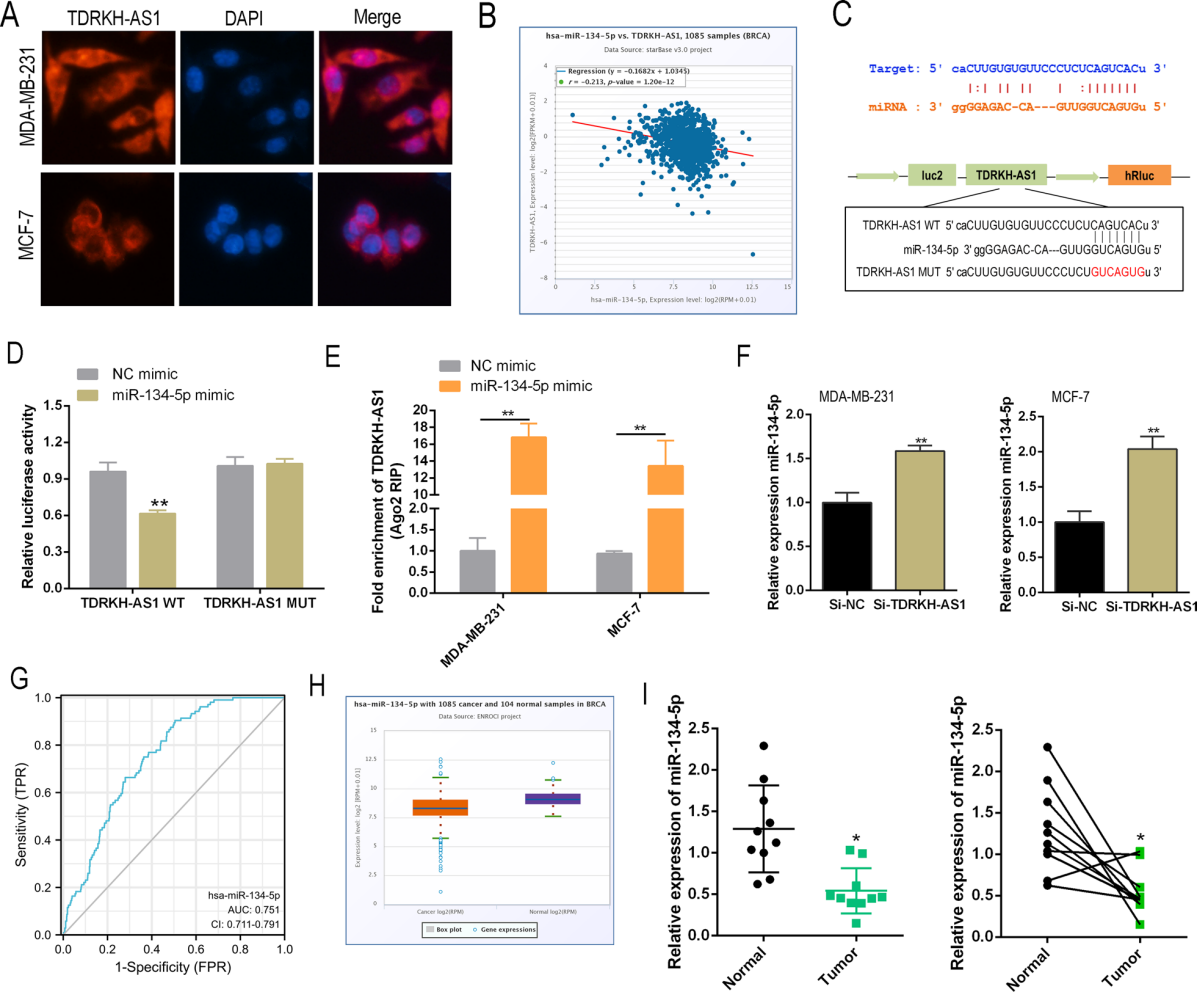
TDRKH-AS1 could act as a ceRNA by binding to miR-134-5p. **A** The cellular localization of TDRKH-AS1 (labeled in red) in breast cancer (BC) cells was determined using RNA fluorescence in situ hybridization (FISH) assay. **B** Spearman correlation analysis revealed a significant negative correlation between TDRKH-AS1 and miR-134-5p in BC cells. **C** In silico prediction analysis suggested that miR-134-5p could potentially bind to TDRKH-AS1. **D**, **E** The interaction between TDRKH-AS1 and miR-134-5p was further confirmed using luciferase reporter assay and RNA immunoprecipitation (RIP) assay. **F** The knockdown of TDRKH-AS1 using siRNA resulted in a significant increase in the expression level of miR-134-5p in BC cells. **G** Receiver operating characteristic (ROC) curve analysis showed that miR-134-5p could be a potential discriminator between BC and normal tissues. **H** The results from the starBase database indicated that miR-134-5p expression was significantly downregulated in BC tissues compared to normal tissues. **I** RT-qPCR was used to detect the expression levels of miR-134-5p in clinical samples, and the results showed a significant difference in expression levels between BC and normal tissues. ^∗^*P* < 0.05; ^∗∗^*P* < 0.01

It is well-established that lncRNAs act as ceRNAs by binding to and isolating target microRNAs (miRNAs) to modulate the expression of target genes. Our immunohistochemistry (IHC) data revealed that TDRKH-AS1 predominantly localized to the cytoplasm (Fig. 3A). Thus, we hypothesized that TDRKH-AS1 may function as an miRNA sponge to modulate the binding of miRNAs to target mRNAs. Pearson’s correlation analysis revealed a significant negative correlation between TDRKH-AS1 and miR-134-5p in the BC dataset obtained from the TCGA database (Fig. 3B). However, no significant correlation was found between TDRKH-AS1 and the remaining potential miRNAs (Additional file 1: Fig. S1). The predicted binding sequences of TDRKH-AS1 and miR-134-5p are shown in Fig. 3C. The luciferase reporter assay revealed that co-transfection of miR-134-5p mimic and the 3′-UTR of wild-type (WT) TDRKH-AS1 led to a marked reduction in luciferase activity (Fig. 3D), suggesting that TDRKH-AS1 interacted with miR-134-5p. The interaction between TDRKH-AS1 and miR-134-5p was validated in BC cells using a RIP-qPCR assay. These findings were consistent with Pearson’s correlation analysis data obtained from the starBase database (Fig. 3E). Moreover, knockdown of TDRKH-AS1 upregulated the expression of miR-134-5p in BC cells (Fig. 3F). The AUC value of the ROC curve for miR-134-5p was 0.751, indicating a strong discriminative ability for BC (Fig. 3G). Further analysis of the starBase database revealed that miR-134-5p expression was significantly lower in BC tissues (Fig. 3H). RT-qPCR analysis of 10 paired clinical BC specimens also revealed a significant reduction in miR-134-5p expression in BC tissue samples (Fig. 3I).

### Inhibition of miR-134-5p reduces the tumor-suppressive effects induced by TDRKH-AS1 knockdown in BC

**Fig. 4:**
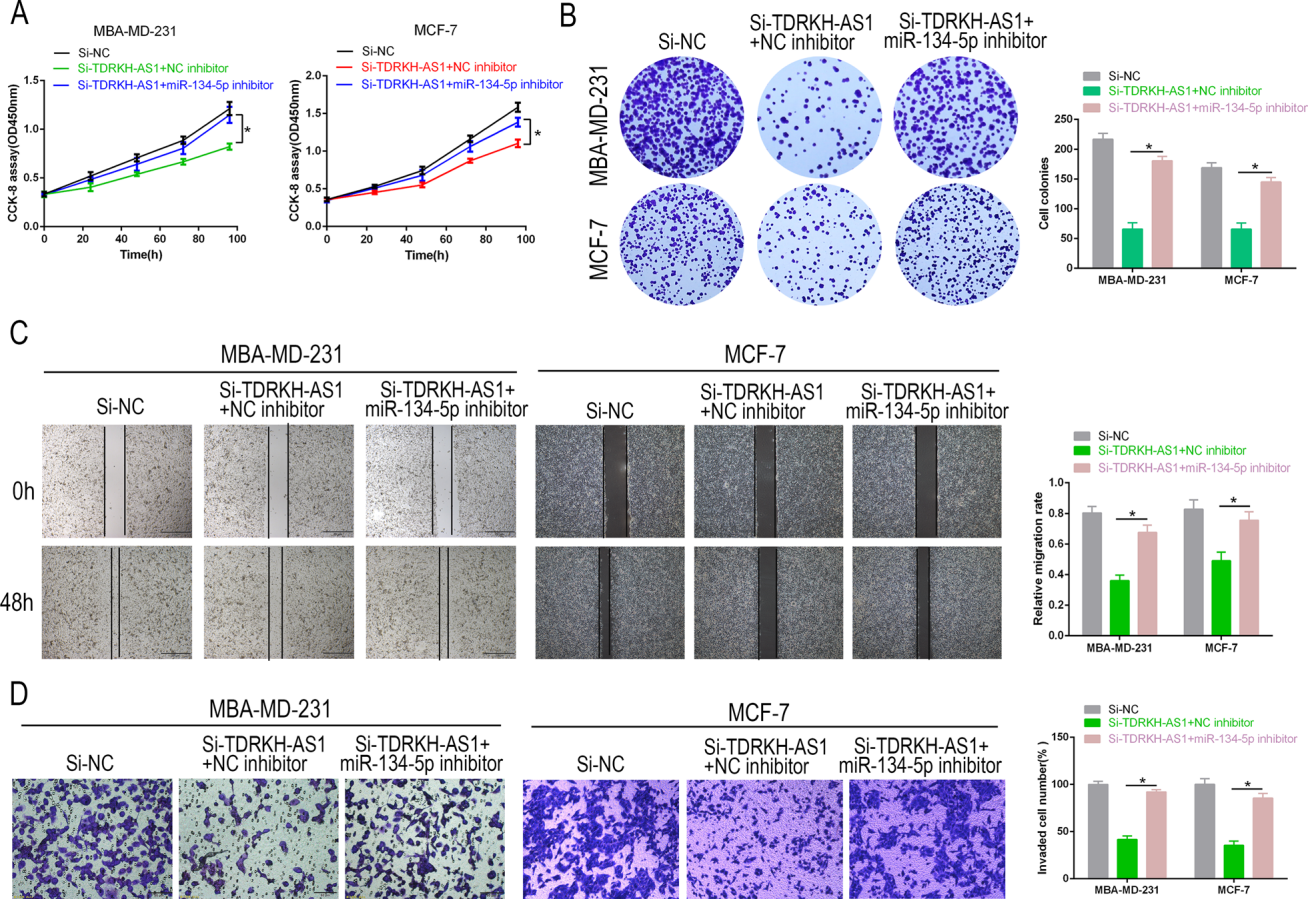
miR-134-5p inhibition reversed the effect of TDRKH-AS1 knockdown on cell proliferation and invasion. **A**, **B** CCK-8 and colony formation assays were used to determine the effect of partial reversal of miR-134-5p inhibitor on the proliferation ability of Si-TDRKH-AS1 in BC cells. **C**, **D** Wound healing and Transwell assays were performed to evaluate the effect of partial reversal of miR-134-5p inhibitor on the migration and invasion of Si-TDRKH-AS1 in BC cells. ^∗^*P* < 0.05; ^∗∗^*P* < 0.01

Next, we performed rescue experiments to examine the relationship between TDRKH-AS1 and miR-134-5p in BC. We found that knockdown of TDRKH-AS1 in the presence of NC inhibitors led to increased miR-134-5p expression and decreased proliferation and invasion. However, inhibition of miR-134-5p was found to partially reverse the tumor-suppressive effects induced by knockdown of TDRKH-AS1 on BC cell proliferation as measured by colony formation and CCK-8 assays (Fig. 4A, B). Similarly, our Transwell and wound-healing data revealed that while knockdown of TDRKH-AS1 reduced the invasive capability of BC cells, this response was partially reversed in the presence of the miR-134-5p inhibitor (Fig. 4C, D). Taken together, our findings suggested that TDRKH-AS1 facilitated BC progression by targeting miR-134-5p.

### CREB1 is the direct target gene of miR-134-5p

**Fig. 5:**
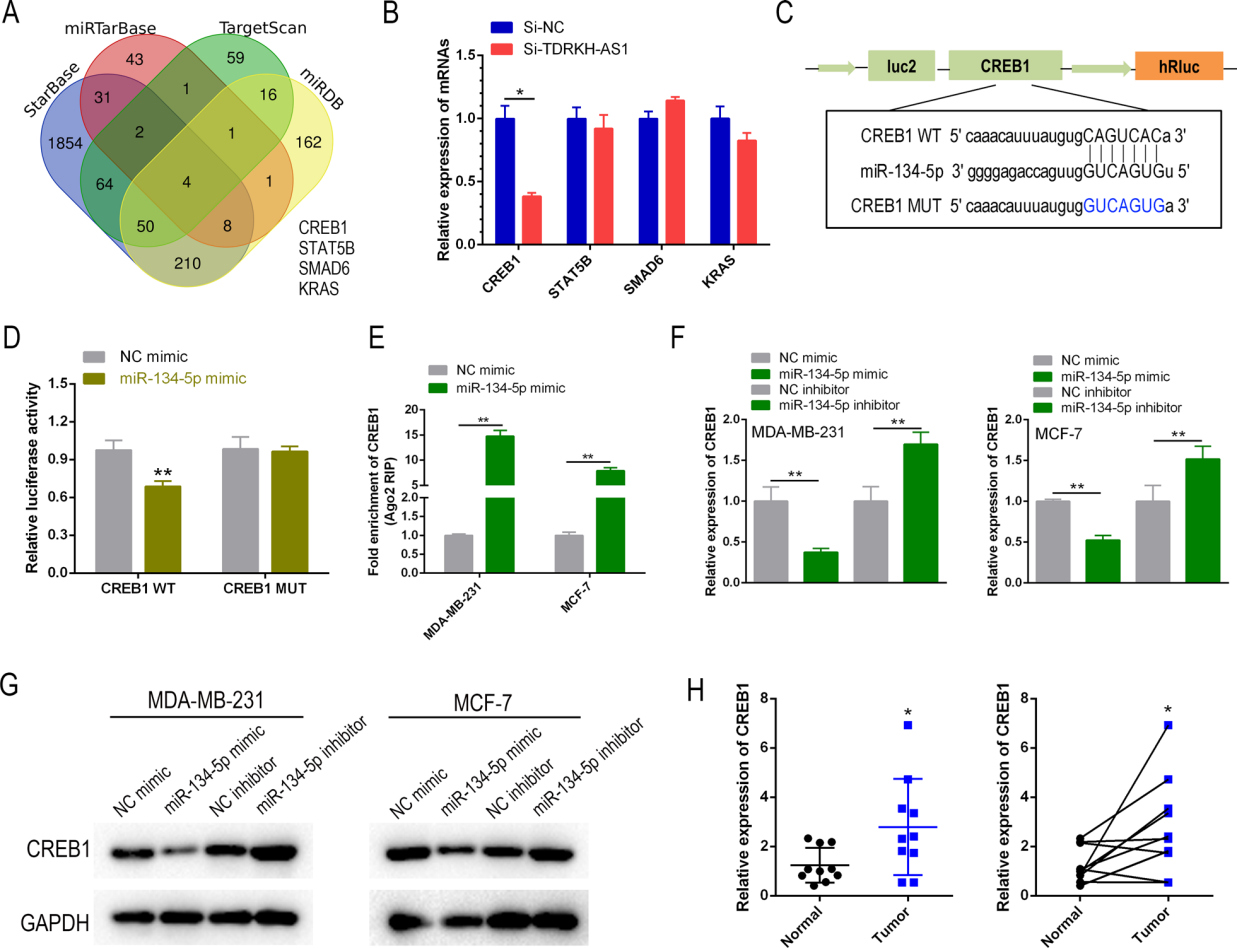
miR-134-5p regulated the expression of CREB1 by targeting it. **A** A Venn diagram was used to identify potential target genes of miR-134-5p in breast cancer, based on predictions from four online databases. **B** RT-qPCR was used to investigate the potential regulatory relationship between TDRKH-AS1 and four potential miR-134-5p target genes in breast cancer cells. **C** In silico prediction analysis suggested that miR-134-5p may bind to CREB1, a transcription factor that plays a role in regulating gene expression. **D**, **E** Luciferase reporter assay and RIP assay were used to confirm the interaction between miR-134-5p and CREB1 in breast cancer cells. **F** RT-qPCR was used to investigate the regulatory effect of miR-134-5p on CREB1 expression in breast cancer cells. **G**, **H** Western blot and RT-qPCR were used to investigate the potential clinical relevance of CREB1 as a target gene of miR-134-5p in breast cancer samples. ^∗^*P* < 0.05; ^∗∗^*P* < 0.01

We identified four potential target genes of miR-134-5p by analyzing various online databases including starBase, TargetScan, miRTarBase and miRDB (Fig. 5A). Of these four potential target genes, CREB1 was the most significantly downregulated in BC cells (Fig. 5B). Further bioinformatics analysis identified a binding site for miR-134-5p in the 3′-UTR of CREB1 mRNA (Fig. 5C). Using a dual-luciferase reporter assay, we confirmed that miR-134-5p bound directly to the 3′-UTR of CREB1. We found that the luciferase activity was significantly lower in CREB1-WT-transfected cells in the presence of miR-134-5p, while no reduction in luciferase activity was observed in the CREB1-MUT group (Fig. 5D). Furthermore, immunoprecipitation assays using the anti-AGO2 antibody demonstrated that endogenous CREB1 mRNA was pulled-down from the BC cell lysates (Fig. 5E). RT-qPCR and western blot analyses revealed that treatment with the miR-134-5p mimic inhibited CREB1 expression, while treatment with the mi-134-5p inhibitor led to a marked increase in CREB1 expression levels (Fig. 5F, G). Furthermore, RT-qPCR analysis of 10 paired clinical BC specimens showed a significant upregulation of CREB1 in BC tissues relative to adjacent non-cancerous tissues (Fig. 5H). In summary, our findings indicated that CREB1 was a downstream target of miR-134-5p in BC cells.

### CREB1 reverses the inhibitory effects of TDRKH-AS1 knockdown on BC cell proliferation and invasion

**Fig. 6:**
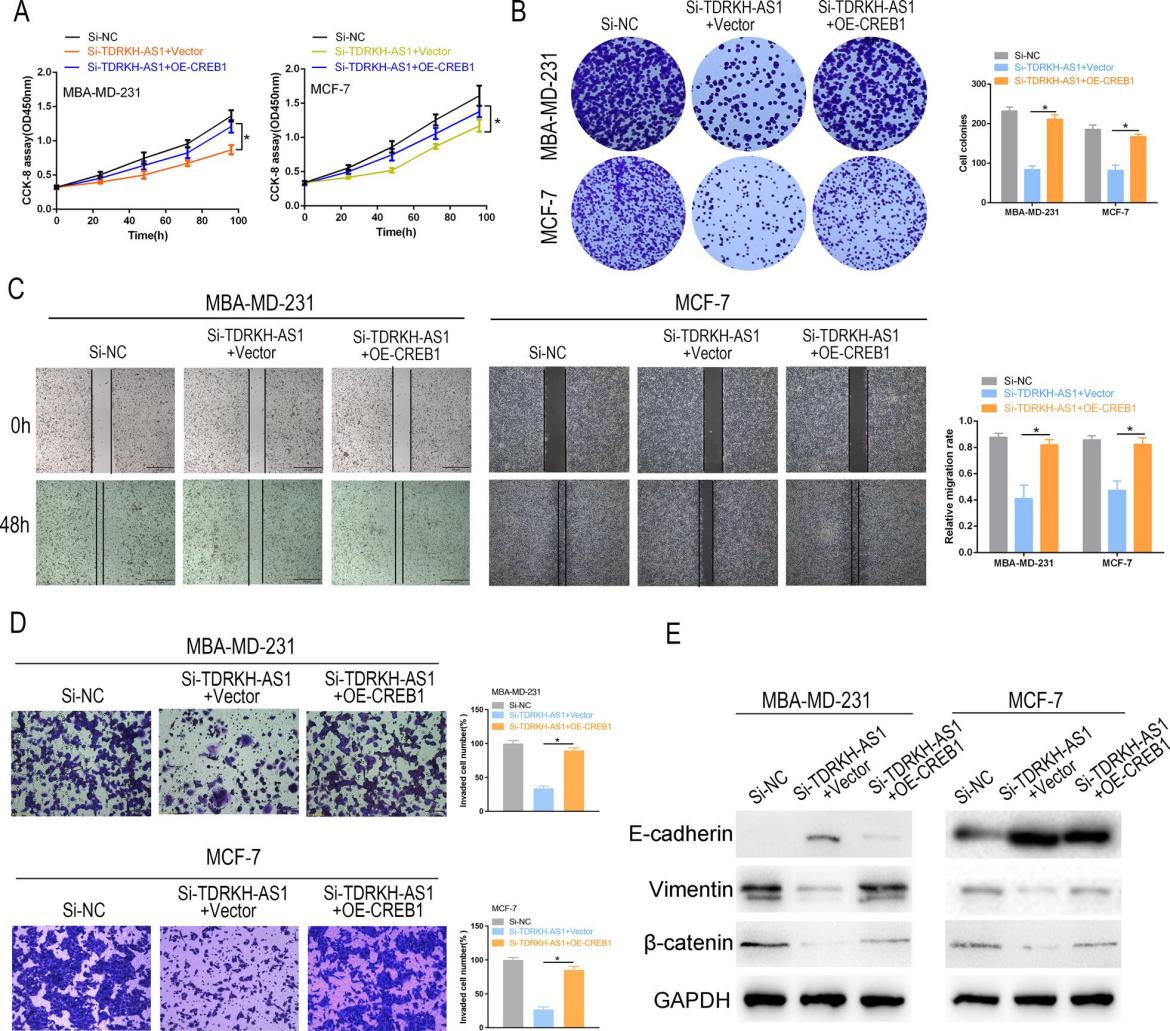
CREB1 reversed the effects of TDRKH-AS1 Knockdown on cell proliferation and invasion. **A**, **B** CCK-8 and colony formation assays were used to determine the effect of partial reversal of OE-CREB1 on the proliferation ability of Si-TDRKH-AS1 in BC cells. **C**, **D** Wound healing and Transwell assays were performed to evaluate the effect of partial reversal of OE-CREB1 on the migration and invasion of Si-TDRKH-AS1 in BC cells. **E** The protein expression levels of E-cadherin, Vimentin, and β-catenin were analyzed by Western blotting in BC cells that were transfected with both Si-TDRKH-AS1 and OE-CREB1. ^∗^*P* < 0.05; ^∗∗^*P* < 0.01

Next, we sought to determine whether the regulatory effects of TDRKH-AS1 on proliferation and invasion were mediated through the CREB1 pathway in BC cells. Overexpression of CREB1 (OE-CREB1) partially restored the si-TDRKH-AS1-induced reduction in cell proliferation as measured by colony formation and CCK-8 assays, suggesting that TDRKH-AS1 regulated cell proliferation through the CREB1 pathway (Fig. 6A, B). Moreover, we found that knockdown of TDRKH-AS1 significantly attenuated cell invasiveness, while overexpression of CREB1 partially reversed this effect, as measured by wound healing, Transwell and western blot assays (Fig. 6C–E). Together, our results indicated that TDRKH-AS1 promoted BC progression through activation of the CREB1 pathway.

### *Knockdown of TDRKH-AS1 inhibits BC tumor growth *in vivo

**Fig. 7:**
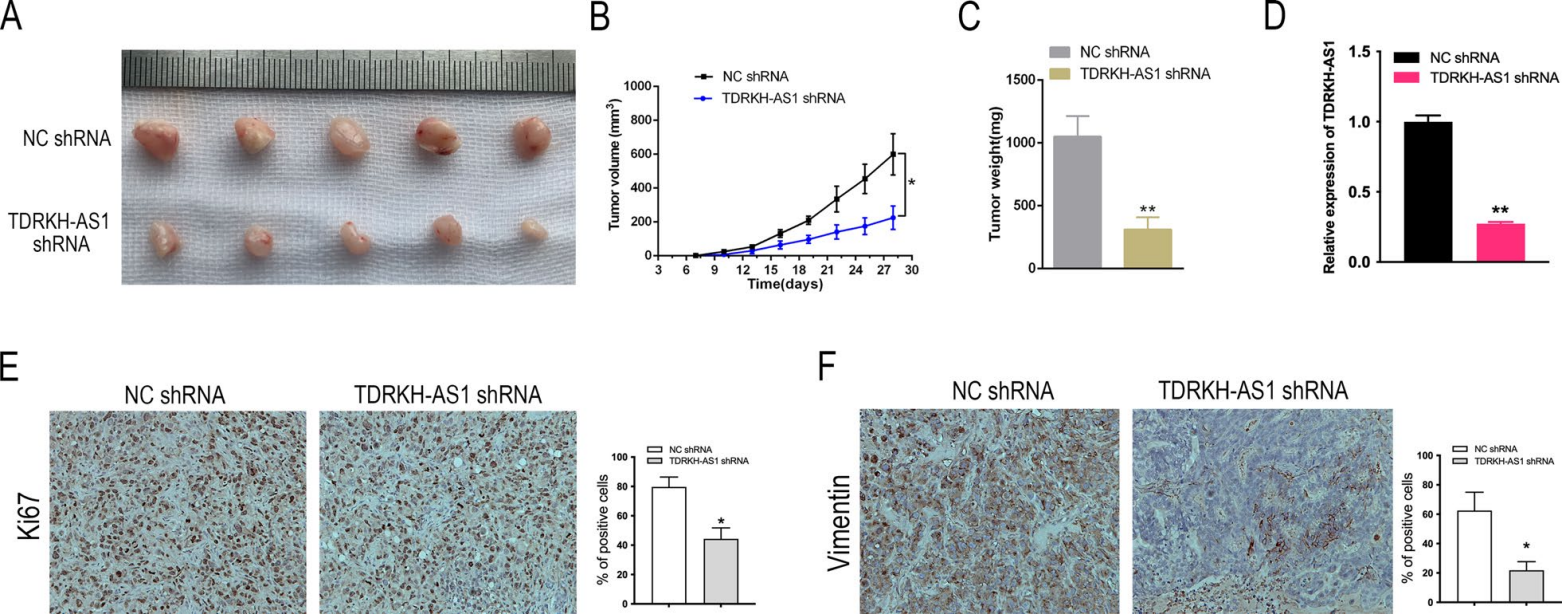
TDRKH-AS1 promoted the tumor growth of BC in vivo. **A** Images of xenograft tumors formed by injection of MDA-MB-231 cells transfected with TDRKH-AS1 shRNA and NC shRNA lentivirus into mice. **B** The average tumor diameter for each week was calculated and presented. Statistical analysis using ANOVA showed that the difference was significant. **C** The weight of the tumors was measured and analyzed using student's t-test. The results showed that the difference was significant. **D**, **E**, **F** Immunostaining analysis was conducted to evaluate the positive rate of Ki67 and vimentin in xenograft tissues. ^∗^*P* < 0.05; ^∗∗^*P* < 0.01

Finally, we examined the role of TDRKH-AS1 in BC tumorigenesis in vivo by inoculating nude mice with MBA-MD-231 cells stably transfected with TDRKH-AS1 shRNA. We found that knockdown of TDRKH-AS1 significantly reduced tumor growth, as measured by tumor morphology, growth curves, and tumor weight (Fig. 7A–C). IHC staining of Ki-67 revealed significantly lower Ki-67 expression levels in subcutaneous tumors formed by TDRKH-AS1-silenced MBA-MD-231 cells than in the control group (Fig. 7D). Furthermore, TDRKH-AS1 knockdown led to a significant reduction in vimentin expression levels (Fig. 7E). These findings suggested that TDRKH-AS1 knockdown impaired BC tumor growth in vivo.

## Discussion

LncRNAs are a class of RNA molecules that have been shown to act as crucial regulators of gene expression and cellular processes [15, 16]. In recent years, lncRNAs have emerged as promising therapeutic targets for cancer treatment [17, 18]. In the current study, we focused on the lncRNA TDRKH-AS1 and its role in BC progression. We found that TDRKH-AS1 was significantly upregulated in both BC tissues and cell lines. Furthermore, our findings indicated that TDRKH-AS1 played a pivotal role in promoting cell proliferation and invasion. Mechanistically, we showed that by acting as a sponge for miR-134-5p, TDRKH-AS1 led to the upregulation of CREB1.

Our findings are consistent with the findings of previous studies examining the role of TDRKH-AS1 in tumor progression. A recent study in HCC found that TDRKH-AS1 was upregulated and promoted tumor metastasis by regulating cell migration and invasion [14]. In LUAD, Wang et al. identified TDRKH-AS1 as a potential prognostic lncRNA through integrated analyses of its expression and copy number alteration profiles [13]. In CRC, TDRKH-AS1 overexpression was associated with poor patient prognosis and enhanced cancer cell proliferation through regulation of the Wnt/β-catenin signaling pathway [11]. Here, we found that TDRKH-AS1 was upregulated in BC tissues and may play a role in BC progression. We also showed that TDRKH-AS1 expression was correlated with tumor size, stage, or metastasis. Furthermore, we found that TDRKH-AS1 was upregulated in luminal, HER2-positive, and TNBC subtypes of BC, with no significant difference observed among the subtypes. Together, our findings suggested that TDRKH-AS1 may have a broad expression pattern in BC, and could, therefore, act as a potential prognostic biomarker. In vivo, we demonstrated that knockdown of TDRKH-AS1 reduced tumor growth, suggesting a functional role for TDRKH-AS1 in BC progression. LncRNAs are superior to proteins as therapeutic targets due to their reduced toxicity. Furthermore, because lncRNAs do not undergo translation, they have a faster turnover rate and lower expression levels, which may allow for quicker therapeutic effects with lower doses. Our findings suggested that TDRKH-AS1 may have therapeutic potential for treating BC, as well as clinical translational significance.

The miRNAs are small non-coding RNAs that play a crucial role in the regulation of gene expression by interacting with mRNA and causing its degradation or translational repression [19, 20]. In human cancers, differentially expressed miRNAs can act as either tumor suppressors or oncogenes [21, 22]. Dysregulation of miRNAs has been frequently reported in many types of cancer, and thus, miRNAs may act as potential diagnostic and therapeutic targets [23]. miR-134-5p is a well-studied miRNA that has been implicated in a wide range of cellular processes and diseases, including cancer [24–27]. Studies have shown that miR-134-5p functions by targeting Yin Yang 1 (YY1) signaling, which can alter the growth and apoptosis of gastric cancer (GC) cells, thereby highlighting its potential as a therapeutic target for GC [28]. Furthermore, exosomal miR-134-5p has been shown to suppress BC proliferation and invasion, and promote apoptosis by targeting ARHGAP1 and inhibiting the PI3K/AKT signaling pathway [29]. Interestingly, overexpression of miR-134-5p has been shown to promote metastasis and chemoresistance of early-stage LUAD cells, indicating that miR-134-5p does not always act as a tumor suppressor in all types of tumors [30]. Our study identified CREB1 as a new target of miR-134-5p and demonstrated that miR-134-5p inhibition by TDRKH-AS1 led to CREB1 upregulation and BC progression.

CREB1 is a transcription factor protein encoded by the CREB1 gene in humans [31]. CREB1 belongs to the leucine zipper family and is known to play a crucial role in the regulation of gene expression by binding to the cAMP response element (CRE) located in the promoter region of target genes [32]. CREB1 is involved in a range of biological processes such as cell survival, differentiation and synaptic plasticity [33]. Dysregulation of CREB1 has been implicated in various types of cancer including BC. Upregulation of CREB1 has previously been associated with poor prognosis and enhanced cell proliferation and migration in BC [34]. Here, we identified CREB1 as a downstream target of TDRKH-AS1 and demonstrated that overexpression of TDRKH-AS1 led to upregulation of CREB1 and BC progression through the miR-134-5p/CREB1 pathway.

In conclusion, our study demonstrated a role for the lncRNA TDRKH-AS1 in promoting BC progression through the miR-134-5p/CREB1 pathway both in vitro and in vivo. The highly invasive triple-negative MDA-MB-231 cell line was used in our in vivo experiments. Thus, it should be noted that while this cell line is a suitable model for studying BC metastasis, the results obtained from this cell line in the current study may not fully represent the in vivo behavior of all subtypes of BC. Our findings have provided novel insights into the mechanisms underlying BC progression and highlight the potential of TDRKH-AS1 as a therapeutic target for BC treatment. Further investigations are needed to determine the clinical significance of TDRKH-AS1 expression and explore its feasibility as a therapeutic target for BC.

## Materials and methods

### Clinical samples and cell culture

BC tissues and matched adjacent normal tissues were collected from 10 patients who received primary BC surgery between May 2021 and October 2022 at the Breast Surgery Department of Zhejiang Cancer Hospital. All patients were diagnosed with BC based on pathological examination and had not received radiotherapy or chemotherapy prior to surgery. Clinical samples were collected immediately after resection, frozen using liquid nitrogen, then stored at – 80 ℃. The study was approved by the Ethics Committee of Zhejiang Cancer Hospital, and written informed consent was obtained from all patients who participated in the study. BC cell lines including CAL-51, BT-549, MBA-MD-231, and MCF-7, as well as one normal epithelial cell line, MCF-10A, were obtained from ATCC for this study. All cells were cultured in RPMI 1640 medium containing 10% fetal bovine serum (FBS) and maintained at 37 ℃ in a humidified incubator with 5% CO_2_.

### Bioinformatics analysis

Expression data and clinical information relating to TDRKH-AS1 were extracted from the TCGA-BRCA dataset of the TCGA database (https://cancergenome.nih.gov/) and analyzed using R software. The starBase database (https://rnasysu.com/encori/) was used to determine the expression of TDRKH-AS1 in BC. The UALCAN database (https://ualcan.path.uab.edu/) was used to determine the expression of TDRKH-AS1 in different BC molecular subtypes including luminal, HER2-positive and TNBC. The GEO database (GSE156229) was used to analyze the expression data of TDRKH-AS1 in BC tumor and non-cancerous tissues. The GEPIA database (http://gepia.cancer-pku.cn) was used to determine the correlation between TDRKH-AS1 expression levels and BC OS and RFS. The starBase database was used to predict the miRNAs targeted by TDRKH-AS1 and their potential binding sites. Potential mRNAs targeted by miR-134-5p were predicted by overlapping the starBase, TargetScan (http://www.targetscan.org/), miRTarBase (https://mirtarbase.cuhk.edu.cn/), and miRDB (http://www.mirdb.org/) data. Further bioinformatics analysis was used to determine the binding site between miR-134-5p and CREB1.

### Cell transfection

The si-TDRKH-AS1, miR-134-5p mimics and inhibitors, and CREB1 overexpression plasmids were purchased from GenePharma (Shanghai, China). si-NC, miR-NC and vector were used as the control groups. BC cell lines were seeded into 6-well plates and cultured for 24 h until they reached a confluency of 60–70%. Then, the cells were transfected with the siRNAs, miRNA mimics and inhibitors and overexpression plasmids using Lipofectamine 2000 (Invitrogen, Carlsbad, USA) according to the manufacturer’s protocol.

### RT-qPCR

The mRNA was reverse transcribed into cDNA using the PrimeScript^™^ RT Kit (TAKARA, Japan) according to the manufacturer’s protocol. The reaction conditions were as follows: 37 ℃ for 15 min, followed by 85 ℃ for 5 s. The cDNA product was immediately used as a template for the PCR reaction. miRNA was reverse transcribed into cDNA using the Mir-X miRNA First-Strand Synthesis Kit (TAKARA, Japan) according to the manufacturer’s protocol. The reaction conditions were as follows: 37 ℃ for 60 min, followed by 85 ℃ for 5 min. The cDNA product was immediately used as a template for the PCR reaction. The TB Green Premix Ex Taq^™^ Kit was used for qPCR analysis according to the manufacturer’s instructions. The reaction conditions were as follows: 95 ℃ for 30 s, followed by 40 cycles of 95 ℃ for 5 s and 60 ℃ for 30 s. GAPDH was used as an internal reference for CREB1 and TDRKH-AS1, while U6 was selected as the internal reference for miR-134-5p. The relative expression levels were calculated using the 2^−ΔΔCT^ method. The primers were synthesized by GenePharma (Shanghai, China). The primer sequences were as follows: miR-324-5p (5′- TGTGACTGGTTGACCAGAGGGG-3′), CREB1 (F: 5′- GACCACTGATGGACAGCAGATC-3′; R: 5′- GAGGATGCCATAACAACTCCAGG-3′), TDRKH-AS1 (F: 5′-ATAGGCTTTGTAGATGGACAGGAAGT-3′; R: 5′-CTCCTGCCGCTGTTTCG-3′), and GAPDH (F: 5′-CCTTCCGTGTCCCCACT-3′; R: 5′-GCCTGCTTCACCACCTTC-3′).

### Western blot

Cells were lysed on ice using RIPA lysis buffer containing protease inhibitors (Beyotime, China). The lysates were centrifuged at 12,000 rpm for 10 min at 4 ℃, and the supernatants were collected. The protein concentration was measured using a BCA Protein Assay Kit (Beyotime, China). Protein samples (40 µg) were separated by SDS–polyacrylamide gel electrophoresis and transferred onto PVDF membranes. The membranes were blocked with milk at room temperature for 2 h, then incubated with primary antibodies against GAPDH (1:2000, CST, USA), E-cadherin (1:1000, CST, USA), vimentin (1:1000, CST, USA), ß-catenin (1:1000, CST, USA), and CREB1 (1:1000, CST, USA) overnight at 4 ℃. The membranes were washed three times with TBST, then incubated with secondary antibodies (1:5000, CST, USA) for 2 h at 4 ℃. The membranes were washed a further three times with TBST, then visualized using a chemiluminescence imaging system (Bio-Rad, USA).

### CCK-8 assay

MDA-MB-231 and MCF-7 cells were transfected with siRNA, miRNA mimics, plasmids, or other agents based on the experimental groups. Transfected cells were seeded into 96-well plates at a density of 5 × 10^4^ cells/mL (100 μL; 6 replicates for each group) and cultured at 37 ℃ in an atmosphere of 5% CO_2_ for 24, 48, 72, and 96 h. At each time point, 10 μL of CCK-8 reagent was added to each well, and the cells were incubated for a further 3 h. The absorbance of each well was measured at 450 nm using a microplate reader (Bio-Rad, USA).

### Colony formation

MDA-MB-231 and MCF-7 cells were transfected with siRNA, miRNA mimics, plasmids, or other agents based on the experimental groups, then seeded into 6-well plates at a density of 1 × 10^4^ cells/mL. The cells were allowed to form colonies over a 14 day period and the medium was replaced with fresh medium every 3 days. After 14 days, cells were fixed with 4% paraformaldehyde, stained with 0.5% crystal violet, washed with PBS and the number of colonies formed was counted under a microscope. The experiment was performed in triplicate to ensure reproducibility.

### Wound-healing assay

MDA-MB-231 and MCF-7 cells were transfected with siRNA, miRNA mimics, plasmids, or other agents according to the experimental groups, then seeded into 6-well plates at a density of 1 × 10^6^ cells/mL. Once the cells had adhered, they were cultured overnight in serum-free medium. A scratch was made using a pipette tip, and the width of the scratch was recorded. The cells were then cultured for 48 h, and the width of the scratch was measured again to assess cell migration. Images were taken to document the changes in the scratch width over time. The experiment was performed in triplicate to ensure reproducibility.

### Transwell assay

Matrigel was diluted to a final concentration of 5 μg/μL and placed in the upper chamber (50 μL per well) to form a gel. MDA-MB-231 and MCF-7 cells transfected with siRNA, miRNA mimics, plasmids, or other agents based on the experimental groups were resuspended in serum-free medium (1 × 10^6^ cells/mL). Cell suspension (100 μL) was placed in the upper chamber, while medium containing 10% FBS (700 μL) was placed in the lower chamber. The cells were incubated for 24 h, then the transwell insert was removed. The cells were washed twice with PBS, fixed with 4% paraformaldehyde for 10 min, stained with 0.1% Crystal Violet for 10 min, then washed twice with PBS. A cotton swab was used to remove cells on the upper surface. Cells that had attached to the lower surface were visualized under a microscope (Nikon, Japan) and photographed.

### AGO2-RIP assay

RIP assays were carried out using the Magna RIP RNA-Binding Protein Immunoprecipitation Kit (Millipore, Germany) according to the manufacturer’s instructions. Briefly, anti-IgG antibody, anti-AGO2 antibody (CST), and magnetic beads were coupled, and the beads were incubated with the corresponding cell lysate overnight at 4 ℃. After protein digestion with proteinase K (Sigma-Aldrich, USA), RNA was extracted using TRIzol (TAKARA, Japan). The expression of TDRKH-AS1 was measured by reverse transcription and RT-qPCR.

### Luciferase reporter assay

The dual-luciferase reporter gene assay was used to confirm the regulatory relationship between lncRNA TDRKH-AS1 and miR-134-5p, as well as CREB1 and miR-134-5p. Fluorescence reporter vectors were constructed by amplifying the wild-type (WT) and mutant (MUT) sequences of TDRKH-AS1 and CREB1 via PCR and inserting them into pmirGLO vectors (pmirGLO-TDRKH-AS1-WT, pmirGLO-TDRKH-AS1-MUT; pmirGLO-CREB1-WT, pmirGLO-CREB1-MUT). HEK-293 T cells were seeded in a 24-well plate and cultured for 24 h. Then, HEK-293 T cells were co-transfected with miR-134-5p mimics, mimic NC, pmirGLO-TDRKH-AS1-WT, pmirGLO-TDRKH-AS1-MUT, pmirGLO-CREB1-WT, or pmirGLO-CREB1-MUT for 48 h. Next, the culture medium was removed, and the cells were washed with cold PBS. The Dual-Luciferase^®^ Reporter Assay System (Promega, USA) was used to measure luciferase activity. Data are presented as the ratio of Firefly to *Renilla* luciferase activity, and at least three independent experiments were carried out.

### *Fluorescence *in situ* hybridization (FISH) assay*

FISH was performed using a lncRNA FISH probe mixture (RiboBio, China) to determine the localization of TDRKH-AS1 in MDA-MB-231 and MCF-7 cells. MDA-MB-231 and MCF-7 cells were seeded into 24-well plates at a density of 5 × 10^4^ cells/mL (400l) and incubated for 24 h. Cells were washed with PBS and fixed with 4% paraformaldehyde. After blocking with 0.5% Triton X-100, samples were incubated with pre-hybridization solution, then hybridized with TDRKH-AS1 probes overnight at 37 ℃. Cell nuclei were stained with DAPI and visualized by fluorescence microscopy.

### In vivo* tumor xenograft model*

Female BALB/c (nu/nu) mice aged 4 weeks and weighing 20–24 *g* were purchased from Jiangsu Jicuiyaokang Biotechnology Co., Ltd. (Jiangsu, China). The mice were housed in a sterile SPF room at a controlled temperature (24 ± 2 ℃) with ad libitum access to food and water. MDA-MB-231 cells were transfected with TDRKH-AS1 shRNA lentivirus. The transfected cells were harvested, resuspended in PBS, and subcutaneously injected (0.2 mL/mouse) into the mice (n = 5 per group). Tumor growth was monitored over a period of 4 weeks by measuring the length (a) and width (b) of the tumors using a caliper, and the tumor volume was calculated using the formula (a × b^2^)/2. After 4 weeks, the mice were humanely killed, and the tumors were collected, weighed, and stored at – 80 ℃ for further analysis. Tumor samples were also embedded in paraffin for histological analysis.

### Immunohistochemistry (IHC)

Mouse tumor xenograft tissues were fixed in 10% formalin, embedded in paraffin, and cut into 4 μm sections. Sections were then subjected to deparaffinization with xylene and dehydration with a series of ethanol gradients. For IHC analysis, sections were incubated with 3% H_2_O_2_ to inactivate endogenous enzymes, then boiled in 0.01 M citrate buffer (pH = 6.0) using a microwave oven. After blocking with 5% BSA solution, sections were incubated with primary antibodies against Ki-67 and vimentin overnight at 4 ℃, followed by incubation with a secondary antibody. Next, samples were stained with DAB and counterstained with hematoxylin. Finally, the sections were dehydrated and sealed in neutral resin. Images were captured using an optical microscope.

### Statistical analysis

All data are presented as mean ± standard deviation (SD). A two-tailed paired Student’s t-test was used to compare two groups and one-way analysis of variance (ANOVA) was used to compare three or more groups. Statistical analysis and graph plotting were carried out using GraphPad Prism 8 and SPSS 20.0 software. Survival was assessed using the Kaplan–Meier method and survival curves were compared using the log-rank test. ^*^P < 0.05, ^**^P < 0.01, and ^***^P < 0.001 were considered to be statistically significant. Each experiment was conducted independently at least three times.

### Supplementary Information

**Additional file 1: Figure S1. **Spearman correlation analysis revealed the correlation between TDRKH-AS1 and potential miRNA targets.

## Data Availability

The datasets used and/or analyzed during the current study are available from the corresponding author upon reasonable request.
