# Supplementary figures and images for: LncRNA TDRKH-AS1 promotes breast cancer progression via the miR-134-5p/CREB1 axis
Source: J Transl Med. 2023 Nov 26;21:854. doi: 10.1186/s12967-023-04640-3 (PMC10676586; doi:10.1186/s12967-023-04640-3)

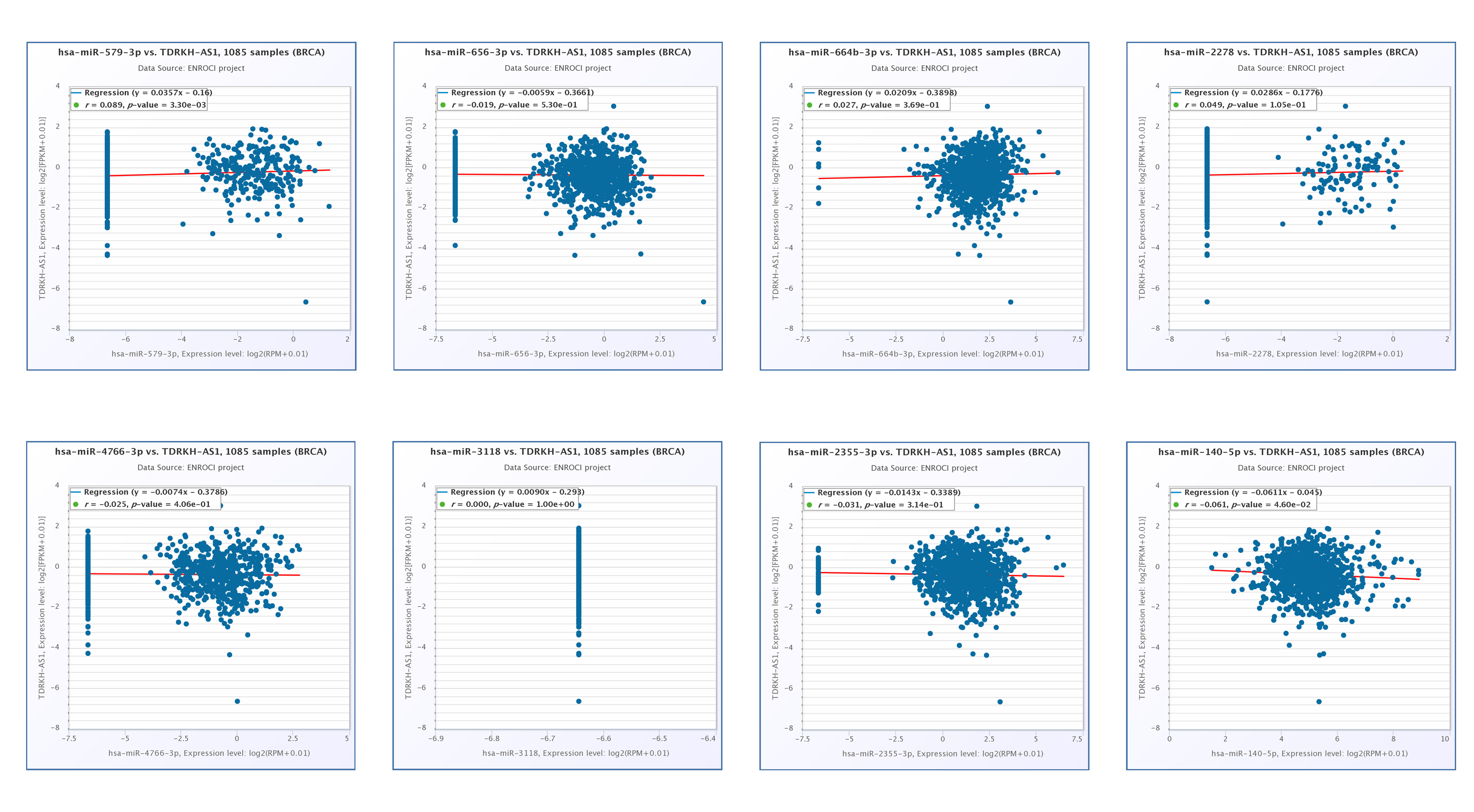

Supplement: Supplementary file 1 — Additional file 1: Figure S1. Spearman correlation analysis revealed the correlation between TDRKH-AS1 and potential miRNA targets. [file 12967_2023_4640_MOESM1_ESM.tif]
